# Genetic determinants of statin intolerance

**DOI:** 10.1186/1476-511X-6-7

**Published:** 2007-03-21

**Authors:** Jisun Oh, Matthew R Ban, Brooke A Miskie, Rebecca L Pollex, Robert A Hegele

**Affiliations:** 1 Schulich School of Medicine and Dentistry, University of Western Ontario and Vascular Biology Research Group, Robarts Research Institute, London, Ontario, Canada N6A 5K8

## Abstract

**Background:**

Statin-related skeletal muscle disorders range from benign myalgias – such as non-specific muscle aches or joint pains without elevated serum creatinine kinase (CK) concentration – to true myositis with >10-fold elevation of serum CK, to rhabdomyolysis and myoglobinuria. The genetic basis of statin-related muscle disorders is largely unknown. Because mutations in the *COQ2 *gene are associated with severe inherited myopathy, we hypothesized that common, mild genetic variation in *COQ2 *would be associated with inter-individual variation in statin intolerance. We studied 133 subjects who developed myopathy on statin monotherapy and 158 matched controls who tolerated statins without incident or complaint.

**Results:**

*COQ2 *genotypes, based on two single nucleotide polymorphisms (SNP1 and SNP2) and a 2-SNP haplotype, all showed significant associations with statin intolerance. Specifically, the odds ratios (with 95% confidence intervals) for increased risk of statin intolerance among homozygotes for the rare alleles were 2.42 (0.99 to 5.89), 2.33 (1.13 to 4.81) and 2.58 (1.26 to 5.28) for SNP1 and SNP2 genotypes, and the 2-SNP haplotype, respectively.

**Conclusion:**

These preliminary pharmacogenetic results, if confirmed, are consistent with the idea that statin intolerance which is manifested primarily through muscle symptoms is associated with genomic variation in *COQ2 *and thus perhaps with the CoQ10 pathway.

## Background

Large clinical trials over the past two decades have unequivocally established the benefit of 3-hydroxy-3-methylglutaryl coenzyme A (HMG CoA) inhibitors – or "statins" – in the primary and secondary prevention of coronary heart disease (CHD) [1]. Reduction of plasma low-density lipoprotein (LDL) cholesterol with statins has revolutionized clinical cardiology; this family of drugs is generally well-tolerated, easy to administer, and has very good patient acceptance. However, the potential for adverse effects exists. From clinical trials, the statin discontinuation rate due to adverse events ranges between 1 and 5% [1]. Frequently reported adverse effects include gastrointestinal complaints, rashes, dizziness, pruritus and headache [2]. Statin-related skeletal muscle disorders range from benign myalgias – such as non-specific muscle aches or joint pains without elevated serum creatinine kinase (CK) concentration – to true myositis with >10-fold elevation of serum CK [3]. The incidence of muscle aches and pains with statin monotherapy ranges from 1 to 7%, whereas severe myopathy, defined as >10-fold elevation of serum CK with muscle pains, weakness and tenderness, occurs in up to 0.5% of patients [3]. Life-threatening rhabdomyolysis with muscle necrosis and subsequent electrolyte alterations, myoglobinuria and renal failure is extremely rare [4]. Rhabdomyolysis is frequently related to use of a statin in combination with such agents as fibric acid derivatives, cyclosporine, macrolide antibiotics or antifungal agents [4].

Recently, genomic variations have been evaluated with respect to inter-individual differences in response to statin therapy, both in terms of LDL-lowering and clinical outcomes [5]. A few studies have also reported associations between polymorphisms in candidate genes and decreased adherence to or increased discontinuation of statin drugs [6]. However, the role of genetic polymorphisms in predicting adverse events associated with statin use remains to be established. Potential candidate genes whose products might be determinants of statin intolerance include those encoding enzymes involved in drug metabolism [6], mitochondrial function [7] or ubiquitination [8]. Ubiquinone, also known as coenzyme Q_10 _(CoQ10), is synthesized, together with mevalonate, by all cells. In theory, inhibition of mevalonate synthesis by statins reduces not only the biosynthesis of cholesterol but also of CoQ10 [9]. Indeed, reduced CoQ10 concentrations have been suggested as a potential mechanism for statin-induced myotoxicity [9]. A role for genetics as a potential determinant was recently demonstrated in two siblings with primary CoQ10 deficiency each of whom was homozygous for a missense mutation in the *COQ2 *gene encoding para-hydroxybenzoate-polyprenyl transferase [10], which is the second enzyme in the CoQ10 biosynthetic pathway. Given the association of CoQ10 deficiency with statin-induced myotoxicity [11], we hypothesized that genetic variation in *COQ2 *was associated with inter-individual variation in statin tolerability.

## Results

### Clinical and demographic data

Descriptive baseline clinical features of statin-intolerant patients and controls are shown in Table 1. All study subjects were self-identified as having European geographical ancestry. Cases and controls were well matched for age, sex, smoking status or treatment for diabetes, hypertension or hypothyroidism. There were no differences in baseline serum C-reactive protein concentration. The proportions of statins used that were atorvastatin, rosuvastatin and all other statins were, respectively, 28%, 26% and 46% in the statin-intolerant group and 45%, 36% and 19% in the control group.

**Table 1:** Baseline attributes of study subjects

	statin-intolerant	controls	P-value
Number	133	158	
percent female	36.6%	35.5%	NS
age (years)	57.1 ± 12.1	55.9 ± 11.3	NS
current smoking	20.3%	21.8%	NS
on treatment for diabetes	24.3%	19.7%	NS
on treatment for hypertension	43.7%	39.2%	NS
on treatment for hypothyroidism	13.2%	11.5%	NS
serum thyrotropin (U/L)	4.4 ± 17.3	2.4 ± 2.7	NS
serum C-reactive protein (mg/dL)	5.0 ± 4.6	4.8 ± 5.7	NS
serum creatine kinase (U/L)			
- highest recorded on treatment	621 ± 927	280 ± 426	<0.0001
- lowest recorded off treatment	175 ± 220	120 ± 120	0.0095
- elevated 5× upper normal limit	17.7%	2.5%	<0.0001
- elevated 10× upper normal limit	5.9%	0.6%	0.009
serum asparagine transaminase (U/L)			
- highest recorded on treatment	47.5 ± 32.1	40.9 ± 17.5	0.03
- lowest recorded off treatment	23.8 ± 8.7	23.5 ± 10.6	NS
- elevated 3× upper normal limit	2.9%	0%	0.03
serum alanine transfaminase (U/L)			
- highest recorded on treatment	41.9 ± 26.7	38.1 ± 20.7	NS
- lowest recorded off treatment	32.3 ± 24.5	27.9 ± 15.4	NS
- elevated 3× upper normal limit	1.5%	0%	NS
hospitalized with rhabdomyolysis	1.5%	0%	<0.0001

Two statin-intolerant patients had been hospitalized with rhabdomyolysis: each developed CK > 10,000 U/L within 6 weeks of statin monotherapy and myoglobinuria and transiently increased serum creatinine, but with eventual return to normal limits of renal function. Compared with control subjects, statin-intolerant patients had significantly higher concentrations of serum CK both at baseline and on treatment, with significantly higher proportions of individuals with CK elevated 5- and 10-fold above the upper normal limit. Compared with control subjects, statin-intolerant patients had significantly higher concentrations of serum asparagine transaminase (AST) on treatment but not at baseline, with significantly higher proportions of individuals with AST elevated 3-fold above the upper normal limit. No differences in alanine transaminase (ALT) were seen. There was no systematic correlation among individuals who had elevations in both CK and AST (data not shown).

### Genetic descriptors of study sample

*COQ2 *allele and genotype frequencies are shown in Table 2. The pairwise linkage disequilibrium correlation coefficient between alleles of SNP1 and SNP2 was 0.81 (P < 0.0001), indicating strong linkage disequilibrium. Maximal likelihood haplotype definitions and frequencies are shown in Table 2. The most common haplotype was designated as "haplotype 1" and had an overall frequency of 0.62.

**Table 2:** Genotype and allele frequencies

		statin-intolerant	controls
SNP-1	G/G	65 (48.9)	85 (53.8)
	G/A	53 (40.0)	66 (41.8)
	A/A	15 (11.1)	7 (4.4)
			
	G	68.9	74.7
	A	31.1	25.3
			
SNP-2	C/C	55 (41.4)	69 (43.7)
	C/G	55 (41.4)	76 (48.1)
	G/G	23 (17.3)	13 (8.2)
			
	C	62.1	67.8
	G	37.9	32.2
			
2-SNP haplotypes	1,1	54 (40.6)	67 (41.9)
	1,2	9 (6.8)	17 (10.6)
	1,3	0	2 (1.3)
	1,4	45 (33.8)	60 (37.5)
	2,2	1 (0.8)	1 (0.6)
	2,4	9 (6.8)	5 (3.1)
	3,3	1 (0.8)	0
	3,4	1 (0.8)	0
	4,4	13 (9.8)	8 (5.0)
			
	1	60.9	66.5
	2	7.5	7.5
	3	1.1	0.6
	4	30.4	25.3

### Genetic associations with statin intolerance

Results of genetic association analysis are shown in Table 3. For association analysis of statin-intolerance, *COQ2 *SNP genotypes were tested using both dominant and recessive models for the minor allele. For association analysis, *COQ2 *haplotypes were collapsed into three groups, based on the presence or absence of haplotype 1. Thus, participants in this study had one of three possible diploid summary haplotypes: 1/1, 1/X or X/X, where X refers to any non-1 haplotype. For association analysis, two models were tested, namely dominant and recessive for the non-1 haplotype alleles.

**Table 3:** Genetic associations with statin-induced myopathy

		Odds ratio (95% CI)	P-value
SNP-1	A-dominant	1.19 (0.75 to 1.88)	NS
	A-recessive	2.42 (0.99 to 5.89)	0.047
			
SNP-2	G-dominant	1.10 (0.69 to 1.75)	NS
	G-recessive	2.33 (1.13 to 4.81)	0.019
			
haplotype	non-1 dominant	1.08 (0.67 to 1.72)	NS
	non-1 recessive	2.58 (1.26 to 5.28)	0.007

Under recessive models for each, the *COQ2 *SNP1 and SNP2 genotypes, and also the 2-SNP haplotype all showed significant associations with statin intolerance. Specifically, the odds ratios (with 95% confidence intervals [CIs]) for increased risk of statin intolerance among homozygotes for the rare alleles were 2.42 (0.99 to 5.89), 2.33 (1.13 to 4.81) and 2.58 (1.26 to 5.28) for SNP1 and SNP2 genotypes, and the 2-SNP haplotype, respectively. Under dominant models for each, the *COQ2 *SNP1 and SNP2 genotypes, and 2-SNP haplotypes were not associated with statin intolerance. There was no association between any genotype and either continuous or discrete CK, AST or ALT variables (data not shown). The subjects with rhabdomyolysis were homozygous for the common SNP alleles and also for haplotype 1.

## Discussion

In this preliminary analysis of a small sample of subjects, we found that genetic variation in *COQ2*, as defined by two genotypes and a haplotype derived from the two most informative SNPs of this gene, was significantly associated with statin intolerance. Specifically, homozygotes for rare alleles of SNP1, SNP2 and the 2-SNP haplotype – ~15% of the study sample – had significantly increased odds of statin intolerance, defined primarily through muscle symptomatology. Significant clinical differences between patients with statin intolerance and control subjects included significantly elevated serum CK both on treatment and at baseline, and elevated serum AST on treatment only. Upon review, there is no clear evidence for the use of other medications that could affect muscle function. Furthermore, the amount and intensity of exercise in the two groups was not recorded, although it was unlikely to have been significantly different.

Genetic determinants of statin intolerance include both common SNPs and rare mutations. For instance, a study conducted primarily in a sample of patients with statin-induced myopathy ascertained through neurology clinics indicated that up to 10% of statin-intolerant subjects were heterozygous or homozygous for disease-causing mutations in rare metabolic myopathies, namely carnitine palmitoyl transferase II deficiency and McArdle disease [7]. Furthermore, muscle biopsies from statin-intolerant patients showed that about half had significant qualitative abnormalities in mitochondrial and fatty acid metabolism without a discrete molecular defect [7]. Such findings are consistent with a biological basis for statin myopathy that has a variety of genetic determinants. The findings from the present study indicate that polymorphism in the *COQ2 *gene may be one such cause out of many in the general population. Mechanistic implication of the ubiquinone pathway is further provided by a recent report of altered ubiquinone levels in muscle biopsies from patients on statins [12].

Some have questioned the clinical significance of statin-related side effects. For instance, a recent systemic overview of randomized clinical trials on risks associated with statin therapy appeared to show no excess of myalgias or CK elevations in patients on statins compared to placebo in 74,000 subjects involved in 35 trials [13]. However, the translation of these findings from controlled clinical trials to the "real world" of clinical practice is questionable. There is little doubt that most clinicians who prescribe statin drugs regularly would be aware of these side effects and would consider them to be an important clinical problem that interferes with statin compliance for many patients.

While our findings support a relationship between *COQ2 *genetic variation and statin intolerance, limitations of this study, as with most association studies [14] include: 1) small sample size; 2) absence of a replication sample; 3) absence of a functional consequence of the genetic variants tested; and 4) absence of genetic associations with intermediate quantitative traits, specifically CK on treatment or muscle pathology. Furthermore, the markers studied may have been in linkage disequilibrium with unmeasured functional marker in *COQ2 *or in some proximal gene that was the actual mechanistic basis of the association.

## Conclusion

Thus, in this preliminary analysis, we found that genetic variation in *COQ2*, as defined by two genotypes and a haplotype derived from the two most informative SNPs of this gene, was associated with increased odds of statin intolerance, defined primarily through muscle symptomatology. Clearly, additional mechanistic and genetic studies are required to confirm these observations. But these preliminary pharmacogenetic results are consistent with the idea that statin intolerance which is manifested primarily through muscle symptoms is associated with genomic variation in *COQ2 *and thus perhaps with the CoQ10 pathway.

## Methods

### Study subjects

We studied 291 patients from a single tertiary referral lipid clinic population. Each patient had been prescribed statin monotherapy for management according to national dyslipidemia guidelines [15]. 133 subjects were statin-intolerant with myopathy, defined as having symptomatic muscle weakness, tenderness and/or pain with at least one of 1) medically advised discontinuation of statin medication on at least two occasions; 2) serum CK elevated to >3-fold of the upper limit of normal while on a statin on at least one occasion; and 3) medically diagnosed rhabdomyolysis. Patients who only showed abnormal blood biochemistry without symptomatic myalgia and/or arthralgia were excluded. No patient was concurrently taking a fibric acid derivative, cyclosporine, macrolide antibiotic or antifungal agent. 158 clinic patients were matched for sex and age within 5 years of statin-intolerant patients; controls consisted of patients who had been on at least 10 mg of atorvastatin or rosuvastatin or 20 mg of other statins for ≥1 year with no reported symptoms and normal serum CK concentration. When more than one subject was suitable as control for a statin-intolerant patient, both were included. In addition to collecting medication and clinical data, determinations of serum C-reactive protein at baseline and of serum CK, AST and ALT off- and on-treatment were obtained.

### DNA analysis

Genomic DNA was isolated from whole blood using the Puregene DNA isolation kit (Gentra Systems Inc, Minneapolis, MN). SNP genotyping was performed using validated TaqMan genotyping assays purchased from Applied Biosystems (ABI, Foster City, CA). Although several rare *COQ2 *SNPs have been identified, we chose to focus on two SNPs that were most polymorphic and thus more likely to serve as informative markers for the gene. SNP1 was a synonymous polymorphism in exon 5 (Assay ID C_28947991_10, rs6535454). SNP2 was a non-coding polymorphism in intron 4 (Assay ID C_28947992_10, rs4693075). SNP genotyping was performed using an allelic discrimination assay (TaqMan^® ^SNP Genotyping Assays, ABI, Foster City, CA) using the ABI Prism^® ^7900 HT Sequence Detection System (SDS) and genotypes were read using automated software (SDS 2.3, ABI, Foster City, CA). Reactions were run in 5 uL volumes using an amplification protocol of 95°C for 10 minutes, followed by 50 cycles of 95°C for 15 seconds, then 60°C for 1.5 minutes. Blinded replication of 5% of samples showed no discrepancies between genotypes.

### Statistical analyses

All analyses were performed using SAS version 9.0 (Cary, NC), with a nominal level of significance defined as P < 0.05. Significance of the deviation of SNP genotype frequencies from Hardy-Weinberg equilibrium was assessed using chi-square analysis. Pairwise linkage disequilibrium between *COQ2 *alleles was determined using correlation coefficients as described [16]. Two-site maximal likelihood haplotypes were constructed using PHASE version 2.0 [17]. Dominant and recessive genetic models of association of statin intolerance with either individual *COQ2 *SNP genotypes or *COQ2 *haplotype were evaluated using chi-square analysis.

## Competing interests

The author(s) declare that they have no competing interests.

## Authors' contributions

JO carried out the SNP genotyping, participated in the statistical analysis and manuscript preparation. MRB carried out the statistical analyses. BAM managed and coordinated the clinical records. RLP carried out the SNP genotyping and participated in manuscript editing. RAH conceived of the study, participated in its design and participated in the manuscript preparation. All authors approved the final version of the manuscript.
